# Air pollution from gas refinery through contamination with various elements disrupts semiarid Zagros oak (*Quercus brantii* Lindl.) forests, Iran

**DOI:** 10.1038/s41598-021-04429-8

**Published:** 2022-01-07

**Authors:** Hamed Dadkhah-Aghdash, Hassan Zare-Maivan, Mehdi Heydari, Mohsen Sharifi, Manuel Esteban Lucas-Borja, Ravi Naidu

**Affiliations:** 1 Department of Plant Biology, Faculty of Biological Sciences, Tarbiat Modares University, Tehran, Iran; 2 Department of Forest Sciences, Faculty of Agriculture, Ilam University, Ilam, Iran; 3 Department of Agroforestry Technology and Science and Genetics, Higher Technical School of Advanced Agricultural and Forestry Engineering, Castilla La Mancha University, Albacete, Spain; 4 Global Centre for Environmental Remediation (GCER), Faculty of Science, The University of Newcastle, University Drive, Callaghan, NSW 2308 Australia

**Keywords:** Environmental impact, Plant sciences, Ecology, Environmental sciences

## Abstract

Soils and oak trees (*Quercus brantii* Lindl.) in Zagros forests are suffering from the air pollution caused by the Ilam Gas Refinery. Thus, for the first time, we investigated the contamination level of sulfur and trace elements in these ecosystems. Sampling of soil and tree leaves was carried out in different seasons of 2019 and at different distances from the gas refinery. The results showed that soils and leaves at the various distances compared with control distance (10,000 m) were more affected by the gas refinery. Distance from the pollution source and physicochemical properties of soils were the main factors affecting contamination of soil elements contents. The soils with pollution load indices (PLI) of 4.54 were in the highly polluted category. Sulfur was at highly polluted category in soils and were highly enriched in trees. The trees mainly absorbed studied elements via their aerial organs. Our findings indicated that oak trees with the highest value of metal accumulation index are influence tools for monitoring various elements in the polluted air produced by the gas refinery. It is recommended that the ecosystem components near the refinery be studied to accurately evaluate disorders in the food chain.

## Introduction

Environmental pollution in countries with rapid industrial development is one of the most challenging persistent causes of danger to human health^1^ as well as generating significant direct and indirect adverse effects on plants^2,3^. Factors including air humidity, precipitation, and hot temperatures may exacerbate pollution damage to plants^4^ even further. Plants can absorb air pollutants through roots and bioaccumulate in the leaves and bark of trees^5–7^. Forests are among the most valuable and widespread components of terrestrial ecosystems and are vulnerable to increasing depositions of trace elements in line with increased unsustainable industrial and economic activity^8,9^. For example, the semiarid oak forests of Zagros that are located in the west of Iran and in bordering Iraq are one of these valuable forest ecosystems. In recent years, Ilam Gas Refinery (IGR) was established and continued its relentless activity, soils and Brant oak trees (*Quercus brantii* Lindl.) in forests are subjected to potentially toxic elements (PTEs) and sulfur gases released from its exhaust output throughout the year and across different distances. These atmospheric contaminants are deposited depending on their particle sizes, wind velocity and direction and accumulate directly on canopies and aerial parts of trees. In the long run, the higher concentrations of trace elements become potentially harmful to the environmental components, such as soil in which case its remediation proves costly and cumbersome^10^. Soils are commonly the sink for accumulating trace elements in terrestrial ecosystems networking to other organisms in the food chain and the atmosphere^11^.

Many factors affect the distribution of PTEs in terrestrial ecosystems. For example, the topography of the impacted area, dominant wind velocity and direction, total leaf surface area index and proportion of plant cover, background bedrock, as well as, the particle size of pollutants, the height of exhausts, and pollution load outputs all affect deposition rate on leaves and soils^12^. Physical-structural properties of the exhaust tower of industrial plants affect the Hydrogen Sulfide gas as the major form of sulfur in the atmosphere and trace elements of which are deposited in soils and plants of terrestrial ecosystems, affecting tree health^13^.

PTEs, from either natural or anthropogenic sources, has high stability in the environment and are generally non-degradable; they can be transported in the air or accumulated and deposited in inner layers of it^14^. Soil type, its physical and chemical properties including texture, and the nature of different trace elements are the main factors that affect the accumulation of elements in soils^15^.

Various pollution indices such as the enrichment factor (EF) and the geoaccumulation index (I_geo_) can be used to measure the level of trace elements in the soil^14^. Although many studies have been conducted on levels of trace elements pollution in urban and agricultural soils, only a few have studied the trace elements pollution assessment of forest soils^16^. For example, Koptsik et al.^17^ reported on the adverse effects of air pollution source on forest ecosystems from Russia. Also, sulfur dioxide was the main pollutant emitted by the Jeddah oil refinery in Saudi Arabia, and it adversely affected the surrounding environment^4^. So far, little work has been done on the biomonitoring of trace elements in the ecosystems near the gas refinery and petrochemical plants in Iran^18^.

Ilam Gas Refinery (IGR) was established about 14 years ago in the sub-mountainous region of Zagros oak (*Quercus brantii* Lindl.) forest ecosystems. Many toxic atmospheric pollutants and trace elements are released from the gas refinery in different seasons of the year and across various distances. These contaminants can accumulate directly in aerial organs of trees from the air portion and indirectly via absorbed pollutants in soils and then transformed to tree organs.

Despite the immense importance of the Zagros forest ecosystems to the living of local inhabitants and forest biota, the establishing of refinery serves development purposes without environmental regard. It is usually claimed that IGR is not a polluting plant. But, there is no data to support this claim. We have provided data that gas refinery activities contaminating the environment, and its contamination is dependent on the season and the distance from the source. The duration of the activity, in the long run, will cause accumulative contamination in the ecosystem components and consumers and potentially cause health risks in the food chain. Findings of this original and first time investigated research will raise awareness and allow environmental authorities to enforce regulations more often and monitor air quality regularly. Besides, we provide a simple analysis methodology of soil samples and tree leaves to monitor levels of metal bioaccumulation in the ecosystem components for better management applications and practices. The objectives of this research are (i) Investigation of the season and distance changes from the gas refinery on the sulfur and various trace elements concentrations in soils and oak leaves, (ii) Assessment of sulfur and trace element contamination levels in soils and tree leaves, and (iii) Establishment of environmental contamination data reference in Zagros Forest ecosystems for future use. We hypothesise that the soils and oak trees will be affected by sulfur and various trace elements’ concentration in different seasons and at different distances from the gas refinery.

## Results

### Multivariate analyses of the effect of season and distance on different elements of soil and leaf samples

**Figure 1:**
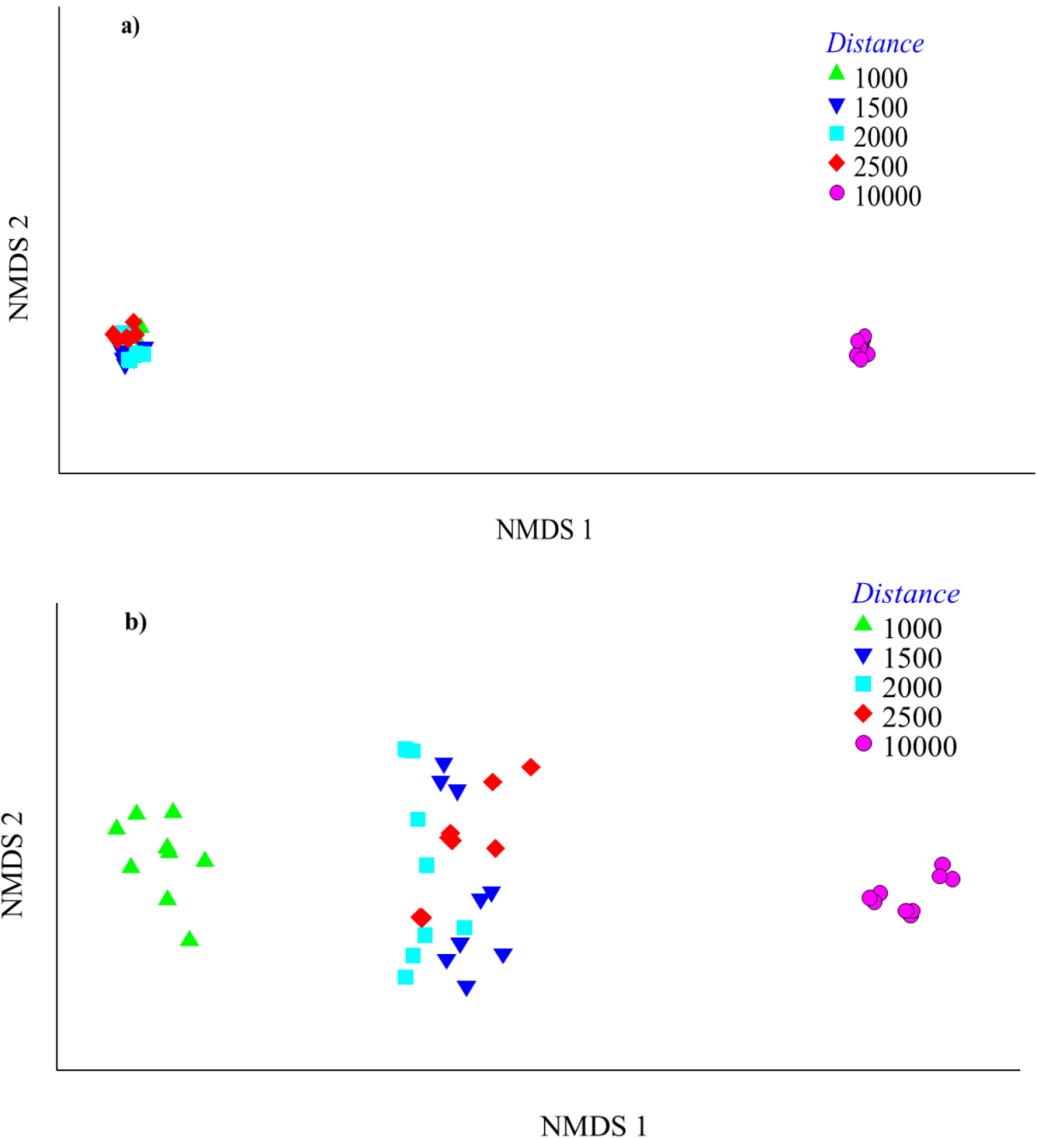
Ordination by soil (**a**) and leaf (**b**) nonmetric multidimensional scaling (NMDS) of distance (1000, 1500, 2000, 2500, 10,000 m) showing the different elements (S, Cr, Cu, Mn, Ni, Pb, As and Zn). Resemblance: S17 Bray Curtis similarity, Transform: Log(X + 1), 2D Stress: 0.01.

**Figure 2:**
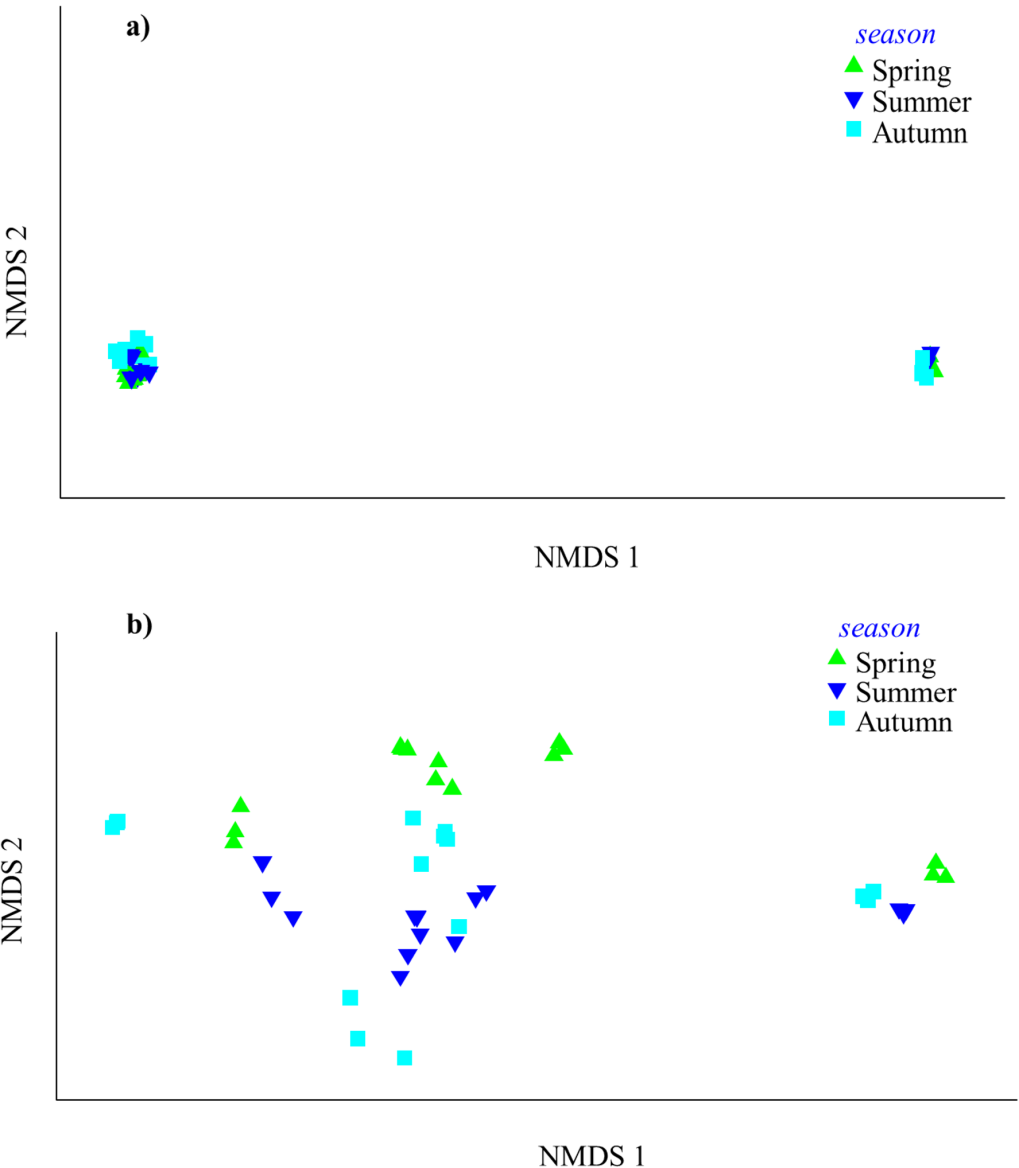
Ordination by soil (**a**) and leaf (**b**) nonmetric multidimensional scaling (NMDS) for season (spring, summer and autumn) showing the different elements (S, Cr, Cu, Mn, Ni, Pb, As and Zn). Resemblance: S17 Bray Curtis similarity, Transform: Log(X + 1), 2D Stress: 0.01.

Regarding multivariate analyses for the soil nonmetric multidimensional scaling (NMDS) for distance (S, Cr, Cu, Mn, Ni, Pb, As, and Zn), there were two clusters: one for distances of 1000, 1500, 2000, and 2500 m (so that different elements at these distances were equally influenced by the pollution source), and the other for the control at 10,000 m distance in a separate cluster. Regarding multivariate analyses for leaf NMDS for distance, there were three different clusters. One cluster was the closest distance (1000 m from the source), another included the 1500, 2000, and 2500 m distances (all of these distances were similarly influenced by pollution), and the third was the 10,000 m (control) distance from the source (Fig. 1a,b). Regarding multivariate analyses for the soil and leaf NMDS for the season (S, Cr, Cu, Mn, Ni, Pb, As, and Zn), no clear pattern was observed for the effect of the season (Fig. 2a,b).

### The effects of air pollution on soil texture and its physicochemical properties

The soil textures at 1000 and 1500 m distances were loam clay; other distances also had a loam texture, with mean clay, silt, and sand percentages of 40.6%, 25%, and 34.4% at these different distances from the IGR plant, respectively.

**Figure 3:**
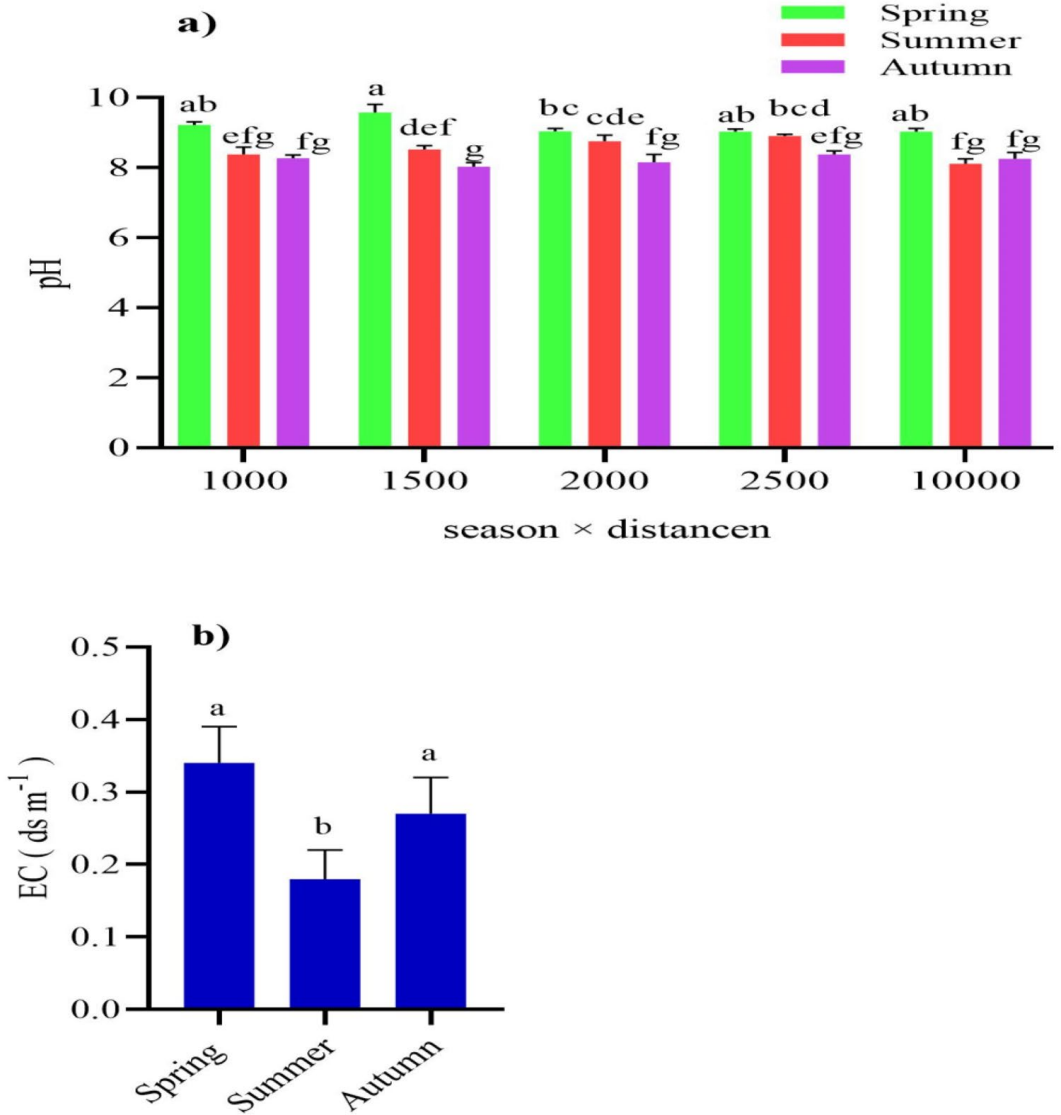
The effects of air pollution on soil properties of pH (**a**) and EC (**b**) for different seasons and distances from the gas refinery.

Soils in summer at the control distance had the significantly lowest basic pH (8.11; p < 0.05) as compared with other distances. Inversely, there were no significant differences in soil pH at various distances in spring and autumn compared with the control distance (Fig. 3a). Soils were with the lowest EC in summer (0.10 ds/m; p < 0.05) compared with the other seasons (Fig. 3b).

### The concentration of different elements in soils and leaves and a comparison

The mean element concentrations of S, Cr, Cu, Mn, Ni, Pb, As, and Zn in the soil samples, in decreasing order, S > Mn > As > Ni > Zn > Cr > Cu > Pb (Table 1). The mean element concentrations (mg kg^−1^) in oak tree leaves were 1,794 for S, 8.07 for Cr, 5.14 for Cu, 22.81 for Mn, 5.3 for Ni, 2.35 for Pb, 11.7 for As, and 26.7 for Zn; thus, in decreasing order S > Zn > Mn > As > Cr > Ni > Cu > Pb. The S and Pb elements in the leaf and soil samples had the highest and lowest concentrations, respectively. The elements of As, Cr, Ni, and S were higher than specified in the soil quality standards of the Iran Department of Environment and world soil standards^19,20^.

**Table 1:** The mean concentration of different elements in soil samples and comparison with world and Iranian soil standard.

Studied element (mg kg^−1^)	Soil	World soil standards	Iranian soil standards
AS	125.02	4.7	17
Cr	53.07	42	0.4
Cu	22.23	14	63
Pb	3.18	25	300
Ni	68.12	18	50
Zn	67.10	62	200
Mn	364.72	418	–
S	7707.69	20	–

### Pollution assessment of different elements in soil and leaf samples

The mean I_geo_ values for S, Cr, Cu, Mn, Ni, Pb, As, and Zn, were in decreasing order and ranked as S > As > Pb > Mn > Ni > Cu > Cr > Zn. The mean soil PI values were 1,457.56 for S, 1.22 for Cr, 1.48 for Cu, 1.73 for Mn, 1.51 for Ni, 3.36 for Pb, 12.35 for As, and 1.06 for Zn; thus, in decreasing order and ranked S > As > Pb > Mn > Ni > Cu > Cr > Zn (Table 2). Besides, the PLIs in all soil samples were 4.54.

**Table 2:** Different pollution indices for S, Cr, Cu, Mn, Ni, Pb, As, and Zn elements in soils and leaf samples of oak trees.

Pollution indices	S	Cr	Cu	Mn	Ni	Pb	As	Zn
PI_Soil_	1457.56	1.22	1.48	1.73	1.51	3.36	12.35	1.06
Igeo_soil_	9.89	− 0.32	− 0.03	0.18	0.0	1.03	2.5	− 0.53
EF_plant_	30.76	1.72	0.24	0.18	1.57	0.79	0.63	0.34
BCF_plant_	0.07	0.13	0.24	0.06	0.07	0.75	0.16	0.40
AOM (%)	75	40	39	32	39	38	40	31

*I*_*geo*_ geoaccumulation index, *PI* pollution index, *BCF* bioconcentration factor, *EF*_*plant*_ enrichment factor of plants, *AOM* air originated metals.

**Figure 4:**
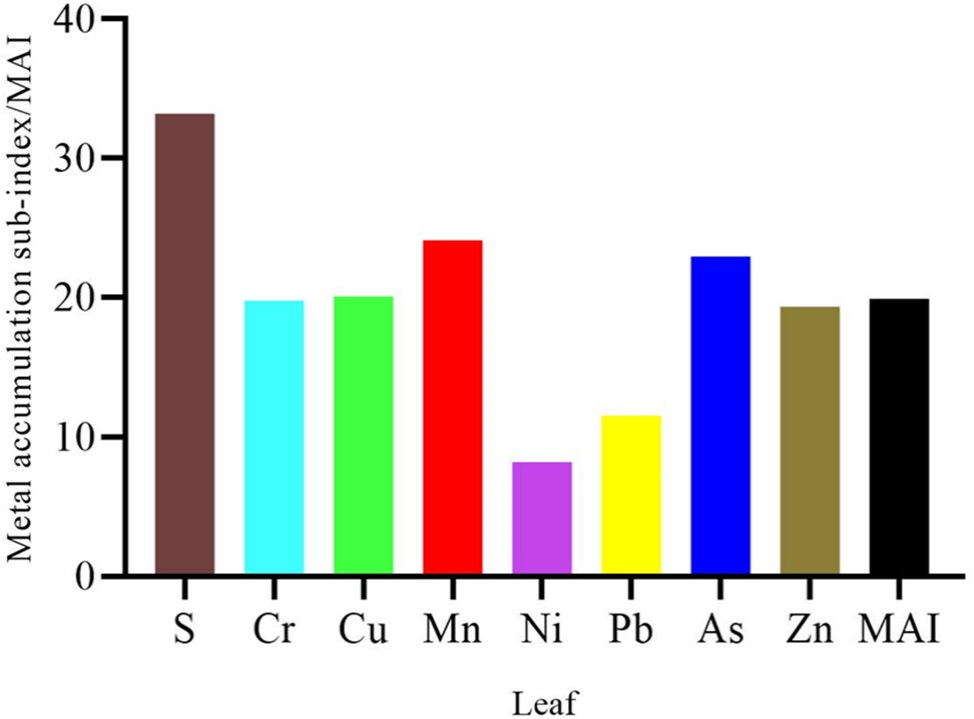
Mean metal accumulation sub-index and MAI of different elements in leaves of oak trees.

The BCF showed the capability of trees to absorb various elements from the soil portion. The mean BCF values for S, Cr, Cu, Mn, Ni, Pb, As and Zn, in decreasing order for accumulation of metals based on the BCF value, Pb > Zn > Cu > As > Cr > S = Ni > Mn (Table 2). The mean EF values for S, Cr, Cu, Mn, Ni, Pb, As, and Zn were in decreasing order, S > Cr > Ni > Pb > As > Zn > Cu > Mn. The mean AOM values were 75 for S, 40 for Cr, 39 for Cu, 32 for Mn, 39 for Ni, 38 for Pb, 40 for As, and 32 for Zn (Table 2). The mean metal accumulation sub-index of studied elements in oak tree leaves was in decreasing order and ranked as S > Mn > As > Cu > Cr > Zn > Pb > Ni. Besides, the MAI of leaves was 19.91 (Fig. 4).

## Discussion

Results indicated pollution loads from the source at different distances affect the concentration of various elements in the soils and leaves differently. Pollution source has differential effects according to how proximate a site is to it. This is evident from the data for distances up to 2500 m from the gas refinery which showed the greatest concentration of trace elements in soil and oak tree leaves. Data analysis clustered control distance separated from the other four distances which clustered together. Lower quantities of elements in soils and leaves at the closest distance are most likely because of the height of the flame exhaust tower of the gas refinery which affects the trajectory of contaminants transport and deposition in conjunction with wind velocity.

Elements in tree leaves were affected by the season and the distance from the source. Due to the semiarid climate of this forest and low available moisture, soil and leaf element loads remained unchanged across seasons. More prevalent spring rains could deposit high pollutants and trace elements that accumulate on the leaf surfaces compared to other seasons, summer was hotter and drier, and autumn lead to leaf senescence. Another broad-leaf forest tree species, such as *Tilia cordata* Mill. had similar responses^21^ to air pollution.

In explaining the concentrations of trace elements in the soil, three sets of factors could be considered: (i) Soil properties such as pH, EC, and texture and (ii) Environmental factors such as distance from pollution source, seasonal changes, and different climatic and geographic conditions and (iii) Physical and structural as well as exhaust output properties of the IGR plant, such as size or height of the exhaust tower and output loads per unit of time. There is a positive correlation with the clay particle size of soil and concentration of trace elements to the highly polluted category in soils^22^. The geology of the bedrock, the geography of the Zagros Forests, and climate conditions in this forest as well as the anthropogenic contribution of the gas refinery caused the sampled soils to have basic pH and differing EC properties across seasons and distances from the gas refinery.

The pH of soils varied by season (moisture and evaporation equation) and distance from the source, but remained in the alkaline range, with an upper limit of 8. The presence of sodium, carbonate and weathered and eroding bedrocks of calcic bedrock matter may be the main reasons that these soils were alkaline^23^. Also, soil samples in summer had the lowest EC compared with other seasons, but overall mean soil EC (salinity) was remained EC < 1 ds/m and no saline condition imposed on plant roots. The findings of this investigation corroborate the results of other researches in the greater Zagros ecosystem that reported similar soil property and behaviour around industrial plants. For example, researchers evaluated soils from different industrial sites in Ahvaz and showed that soils had high pH with basic properties and low EC and were almost entirely clay^24^. Also, Solgi et al.^23^ found that the pH of soils was in the neutral to the alkaline range and had a low EC value that was not saline and the main texture of the soils was loam. In line with our results that soils had pH > 7, and contained mostly clay which contributes to low mobility of ions and trace elements in soils, other researchers found that the pH value of soils causes most ions to have low mobility^25,26^.

The elements of As, Cr, Ni, and S had higher concentrations than recommended by both Iranian and World Soil Quality Standards for the environment. While sulfur as a nutrient plays an important function in the soils of forest ecosystems, greater amounts of it will not benefit these ecosystems^27^. Sulfur concentrations were higher than established normal levels in both soil and leaf samples. Similar results were reached by Cicek and Koparal^19^, who found that soils and oak trees (*Quercus infectoria* L.) around a pollution source in Turkey showed a greater sulfur content than of normal range for soils (0.006–0.02 g kg^−1^) and plants (0.01–0.15 g kg^−1^). Besides, the concentration of studied elements in soil portions was generally higher than those in tree leaves.

Our results show that oak trees had normal ranges for Cu, Zn, and Pb. According to Kabata-Pendias et al.^28^and Padmavathiamma et al.^29^, the concentration of Cu, Zn, and Pb in the ranges of 2–20 mg kg^−1^ and 10–150 mg kg^−1^ and less than 10 mg kg^−1^ are normal.

Soils show different affinities for adsorption of trace elements, as well as tree species, show different affinities for absorbing ions and possibly bioaccumulating trace elements. For example, Serbula et al.^26^ while assessing various heavy metals in *Robinia pseudoacacia* L. trees across different sites, found that Hg, As and Cd were lower than the detection limit and Pb concentration was in the toxic range. In this investigation, As had a higher concentration and Pb was in the normal range in oak tree leaves.

According to Qing et al.^30^ the mean I_geo_ of S fell in the extremely polluted level, As was in the moderately to the extremely polluted level, Pb was in the moderate range, Mn and Ni were considered uncontaminated to the moderately polluted level, and Cr, Zn, and Cu were practically at unpolluted level. The results for I_geo_ and PI showed that S had the highest and Cr, and Zn had the lowest pollution in soil samples. The PLIs in all soil samples were in the highly polluted level.

In this research, oak trees with BCF < 1 for studied elements might do not accumulate metals from soils. Due to alkaline pH and low EC of soils, almost all of the elements had low mobility in soils and low absorption by oak trees^31^. This is similar to findings of Serbula et al.^26^ on the assessment of heavy metals absorption by leaves of *Robinia pseudoacacia* L. trees across different alkaline (pH > 7) sites which showed lower metal mobility in soils and low absorption by trees. Therefore, it is suggested that oak trees accumulate studied elements from aerial portions, primarily through their leaves.

The element of sulfur with EF > 2 was enriched in tree leaves. The properties of leaf organs, such as wax thickness and composition cause different elements to have different enrichment capacities in leaves. These elements could be taken from the air in leaves to enriched level compared to other elements.

The properties of leaves such as wax layers, the surface condition of leaves, and their structures are important factors that determine the attraction of different metals from the air^18^. Oak tree leaves with mentioned properties directly can both absorb and accumulate almost all of the elements with aerial organs. S and Zn had the highest and lowest capacity for accumulation among the studied elements, respectively. In line with our results, Solgi et al.^23^ found that concentrations of Zn, Cu, and Pb in leaves of *Fraxinus excelsior* L. by washing with distilled water decreased and their potential to reduce concentration varied among the metals.

Several factors that determine the accumulation of different elements by aerial organs of trees from the atmosphere include forms and structures of trace elements and their concentration, various tissues and anatomy of tree components, and their reactions and capacities to accumulate trace elements^32^. Oak leaves had various reactions to the pollution of the atmospheric elements of the gas refinery. They were enriched with trace elements from the air portion. Besides, sulfur in leaves had the highest metal accumulation sub-index. Thus, trees mainly accumulate trace elements from the aerial organs. On the contrary to our results, Safari et al.^18^ evaluated various trace elements in ecosystems around the petrochemical industrial sites and found that tree leaves had low MAI.

Zagros ecosystem provides many values and services in the region, including services such as for sheep herds and cattle from tree leaves and grasses as well as wild animal species from grass and oak tree seeds. The health risk is that air pollution from the gas refinery will disrupt these ecosystem components. The concentration of elements was higher in soils relative to tree leaves. The greater hurdle in the long run will come from pH changes affecting, in turn, the survivability of buried plant seeds. In addition, contaminating elements could enter the food chain, through herbaceous plants in growing seasons and potentially place human and other consumers' health at risk^33^. Therefore, to study more precisely the effects of pollution caused by the gas refinery on the food chain, it is recommended that herbaceous plants are grown in these forest ecosystems be studied further.

## Conclusions

Variations in distance from the source compared with different seasons of the year had a higher effect on the concentration of sulfur and trace elements in the soil samples and tree leaves. Some physicochemical properties of soils in Zagros forests induced to various elements had low mobility in the soils, and oak trees almost did not absorb them from the soil portion. According to different pollution indices, sulfur was at the high pollution level in soil samples, oak leaves and was highly aerial accumulation and enriched in tree leaves. It is concluded that soil and leaf samples were contaminated with various elements, especially with sulfur. Contaminating elements in herbaceous plants and tree leaves that grow in polluted soils could enter the food chain in growing seasons, and potentially place human and other consumers’ health at risk.

## Material and methods

### Description of study areas

IGR plant (33° 42/N, 46° 13/E) is located along the edge of the mountains of Zagros forests and 25 km from Ilam city. Its main activity, to supply gas to the western provinces of Iran, started in 2007. It converts sour gas to sweet gas and also produces various products such as pastil sulfur, ethane, and liquefied gas. The refinery has two chimneys, which release waste gases into the atmosphere. Oak trees are the main tree species of the Zagros forests around the refinery; these are exposed to various air pollutants and different elements from this source. Based on random analysis of exhaust emissions, sulfur dioxide and sulfide hydrogen are the major pollutants emitted from the flare gases of this refinery plant^34^. The sampling points have an average altitude of about 1000–1250 m and a slope of less than 20%. The climate of the region is semiarid and influenced by Mediterranean winds. The predominant wind direction was west and southwest. The highest and lowest air temperatures were 41.4 °C and − 11.3 °C, respectively. The average annual rainfall was 71.94 mm (http://www.amarilam.ir).

### Samples collection and analyses

All methods were carried out in accordance with the relevant institutional, national, and international guidelines and legislation. Besides they were discussed and approved by the Research Ethics Committee of Tarbiat Modares University. The formal identification of the *Quercus brantii* Lindl. was performed by H. Dadkhah-Aghdash based on colorful Flora of Iran^35^. The permissions or licenses to collect Brant oak (*Quercus brantii* Lindl.) trees in Zagros forests were obtained. A voucher specimen of Brant oaks were collected and deposited at the Herbarium of department of Plant Biology of Tarbiat Modares University.

**Figure 5:**
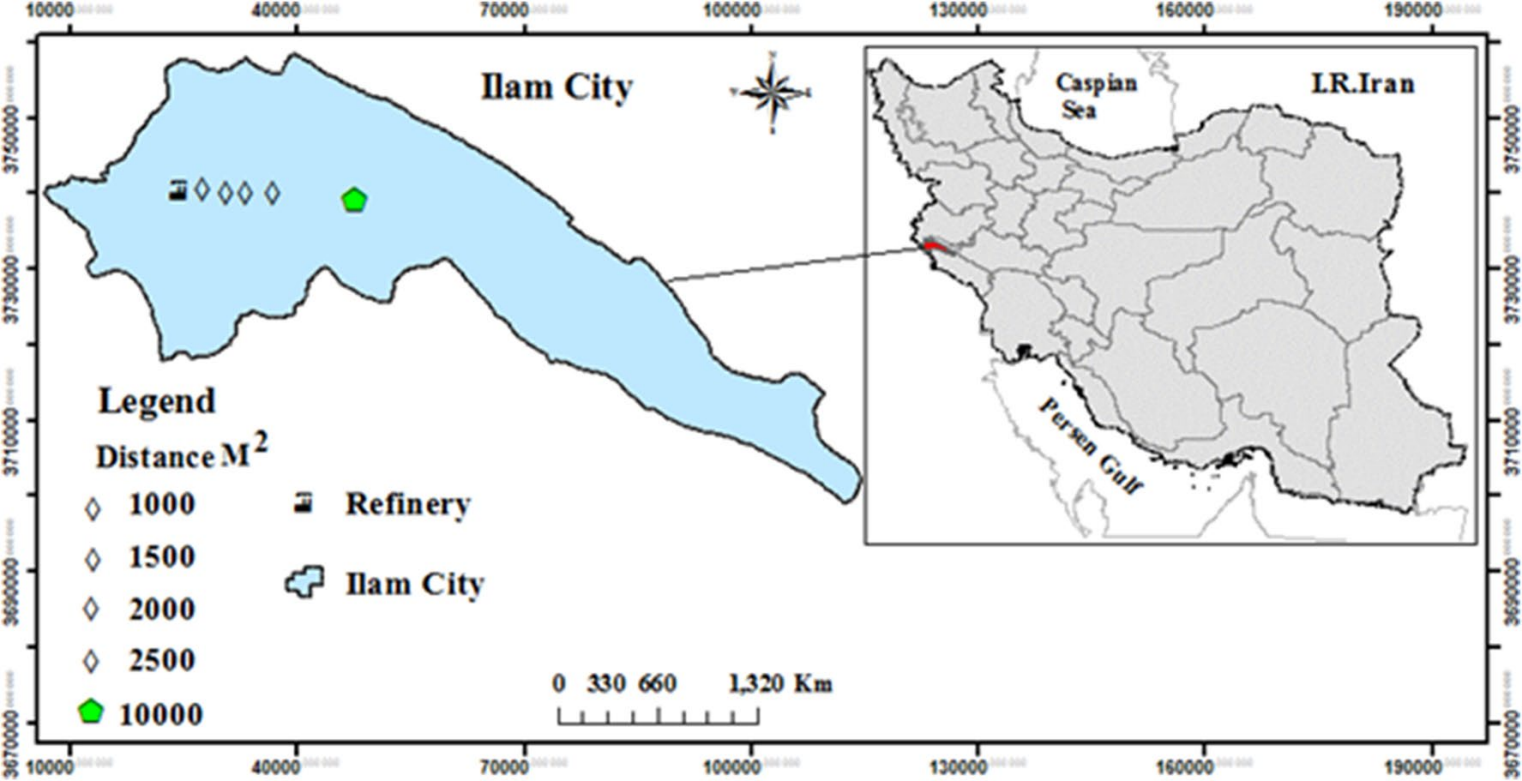
Locations of collection sites of soil samples and Brant oak leaves at five different distances (1000, 1500, 2000, 2500 and 10,000 m [control]) from the gas refinery (drawn by H. Dadkhah-Aghdash using software of ArcGIS Desktop. version of 10.5. ESRI, California, US. https://desktop.arcgis.com).

We studied different distances (1000, 1500, 2000, 2500, and 10,000 m [control]) in an easterly direction from the gas refinery. The map of study area was drawn by software of ArcGIS version of 10.5, https://desktop.arcgis.com (Fig. 5). At each distance, three soil samples taken from the depth of 0–20 cm with a plastic gardening shovel, 30 healthy and mature leaves were collected from a certain height (nearly the middle of the canopy) and the outer canopy of three Brant oak trees in the late spring, summer, and autumn of 2019. These trees with average height and diameter at breast height of 5.5 m and 45 cm were selected randomly. The leaf and soil samples were put into polyethylene bags and transported to the laboratory for analysis^36^.

In the lab, firstly the leaves were categorized into two types: unwashed leaves and leaves washed with ethylenediaminetetraacetic acid (EDTA) solution to remove some atmospheric dusts and particles deposition. The leaf and soil samples were dried for 10 days until they reached a constant weight at lab temperature. The leaves were grinded and homogenized, soils were sieved with ASTM mesh (DAMAVAND, Iran) with a diameter of 2 mm and homogenized.

To determine the pH and electrical conductivity (EC) of soils, 2 g of the soil samples were shaken in 10 ml of double-distilled water with a ratio of 1:5; after 1 h, the pH and electrical conductivity (EC) of the solution were measured by a digital pH meter (Fan Azma Gostar Company, Iran) and EC meter (Sartorius, PT-20, USA). The analysis of the particle sizes of the soil was carried out using the hydrometer method and texture class was determined with a soil texture triangle^37^.

According to different U.S.EPA protocols that were modified by following references, the soil and leaf samples were prepared and dissolved. The digestion of soil samples was conducted with a mixture of concentrated HF–HClO_4_–HNO_3_^38^. Approximately 0.5 g of dry soil sample was digested with 10 mL of HCl on a hot plate at ~ 180 °C until the solution was reduced to 3 mL. Approximately 5 mL of HF (40%, w/w), 5 mL of HNO_3_ (63%, w/w), and 3 mL of HClO_4_ (70%, w/w) were then added and the solution was digested. This process was continued with adding 3 mL of HNO_3_, 3 mL of HF, and 1 mL of HClO_4_ until the silicate minerals had fully disappeared. This solution was transferred to a 25 mL volumetric tube, and 1% HNO_3_ was added to bring the sample up to a constant volume for the element's determinations. After filtering the digested samples, the concentrations of sulfur (S), arsenic (As), chromium (Cr), copper (Cu), lead (Pb), zinc (Zn), manganese (Mn), and nickel (Ni) were measured via inductively coupled plasma mass spectrometry (ICP-MS,7500 CS, Agilent, US). The procedures of quality assurance and quality control (QA/QC) were performed.To quantify element contents from soil samples, external standards with calibration levels were used. The precision and the repeatability of the analysis were tested on the instrument by analyzing three replicate samples.

According to Liang et al.^39^ leaf samples were acid digested and sieved powder samples were placed in the acid-washed tubes and 10 mL of 65% nitric acid was added to it. The solution was placed at room temperature overnight (12 h) after that, it was placed for 4 h at 100 °C and then 4 h at 140 °C until the solution color was clear. After cooling, the solution was diluted by deionized water to 50 mL and then passed through Whatman filter paper until 25 mL of the filtrate volume was provided. Each sample was digested three times and the average of measurements is reported. Total plant elements were measured by using the ICP-MS (7500 CS, Agilent, US). A control sample was also used beside each sample to determine the background pollution during digestion. To confirm the accuracy of the methodology and to ensure the extraction of trace elements from the leaf samples, the standard solution of each studied elements was used.

### Measuring of pollution levels of different elements in soils and leaves

For assessment of contamination levels (concentration) of different elements in soils and trees, common indices of pollution including geoaccumulation index (I_geo_), pollution index (PI), pollution load index (PLI), enrichment factor of plants (EF_plant_), bioconcentration factor (BCF), air originated metals (AOM ), metal accumulation index (MAI) were used.

I_geo_ was calculated using the following (Eq. 1):

1 $${\text{I}}_{{{\text{geo}}}} = \log_{2} \left[ {{\text{C}}_{{\text{n}}} / 1.5{\text{ B}}_{{\text{n}}} } \right]$$

where C_n_ is the measured concentration of the element n, B_n_ is the geoaccumulation background for this element and 1.5 is a constant coefficient used to eliminate potential variations in the baseline data^40^. The I_geo_ classifies samples into seven grades: < 0 for practically unpolluted; 0–1 for unpolluted to moderately polluted; 1–2 for moderately polluted; 2–3 for moderately to strongly polluted; 3–4 for strongly polluted; 4–5 for strongly to extremely polluted; and > 5 for extremely polluted^30^.

The first PI is expressed as (Eq. 2):

2 $${\text{PI }} = {\text{ C}}_{{\text{i}}} /{\text{S}}_{{\text{i}}}$$

where C_i_ is the concentration of element i in the soil (mg kg^−1^) and S_i_ is the soil quality standard or reference value for element i (mg kg^−1^). The PLI for different elements is calculated via the (Eq. 3):

3 $${\text{PLI}} = \left( {{\text{PI}}_{{1}} \times {\text{ PI}}_{{2}} \times {\text{ PI}}_{{3}} \times \cdots \times {\text{PI}}_{{\text{n}}} } \right)^{{{1}/{\text{n}}}}$$

The PLI of soils is classified as follows: PLI < 1 is unpolluted, 1 < PLI < 2 is unpolluted to moderately polluted, 2 < PLI < 3 is moderately polluted, 3 < PLI < 4 is moderately to highly polluted, 4 < PLI < 5 is highly polluted, and PLI > 5 is very highly polluted.

Plant EF is calculated as (Eq. 4):

4 $${\text{EF}}_{{{\text{plant}}}} = {\text{C}}_{{{\text{plant}}}} /{\text{ C}}_{{{\text{control}}}}$$

where C_plant_ and C_control_ are element concentrations (mg kg^−1^) in tree leaves at the polluted site and the control site, respectively. A value of EF > 2 indicates that a tree is enriched with the specific element^41^.

BCF, which indicates the ability of plants to accumulate different elements from the soil^7,42^, is calculated as (Eq. 5):

5 $${\text{BCF }} = \left( {\left[ {\text{C}} \right]_{{\text{L}}} / \left[ {\text{C}} \right]_{{\text{S}}} } \right)$$

where [C]_L_ and [C]_S_ are, respectively, the concentration of different elements in leaf and soil samples. Values of BCF > 1, BCF < 1, and BCF = 1 imply that the trees are accumulators, excluders, and indicators, respectively, for different elements^39^.

AOM, used to determine the number of different elements in leaves originating from ambient air^18^, are calculated by the (Eq. 6):

6 $${\text{AOM }}\left( \% \right) = \left( {\left( {{\text{C}}_{{{\text{unwashed}}\;{\text{leaves}}}} } \right) - \left( {{\text{C}}_{{\text{washed leaves}}} } \right)} \right) / \left( {{\text{C}}_{{\text{unwashed leaves}}} } \right) \, * \, 100$$

where (c) is the concentration of studied element.

Oak tree leaves had different abilities to accumulate atmospheric studied elements. MAI was calculated as (Eq. 7):

7 $$\text{MAI}=1/\text{n}\sum_{\text{j}=1}^{n}\text{Ij}$$

where n is the total number of studied elements, and Ij is the sub-index of variable j, calculated by dividing the mean concentration (x) of each element by its standard deviation^43,44^.

### Data analysis

Before analysis of variance, the normality of the data distribution was checked by SAS version software using Shapiro–Walk and Kolmograph-Smirnov tests. The logarithmic conversion was used to the normality of the data in Excel. Different properties of soils such as pH and EC for two factors including seasons in three levels (spring, summer, and autumn) and distance in five levels (1000, 1500, 2000, 2500, and 10,000) with three replicates were analyzed in a completely randomized design. Analysis of variance was performed by two-way ANOVA with SAS software and the comparisons of means were conducted with Duncan’s test at p < 0.05. The differences among the distance and season factors were studied using a resemblance matrix for the different elements (S, Cr, Cu, Mn, Ni, Pb, As, and Zn). The resemblance is the general term in the PRIMER software used to cover (dis)similarity or distance coefficients between all pairs of the samples.

Following this, elements matrices were log x + 1 transformed, and the resemblance matrix was built using the Euclidean distance. Elements were analysed using nonmetric multidimensional scaling (NMDS) and the Kruskal stress formula (minimum stress: 0.01) for visualizing the level of similarity. Statistical analyses were conducted using PRIMER 6 software^45,46^.

## Data Availability

The data used to support the findings of this study are available from the corresponding author upon reasonable request.
